# Depression in patients with chronic otolaryngology symptoms – A vicious cycle

**DOI:** 10.1186/s13005-024-00464-8

**Published:** 2024-11-15

**Authors:** Meera Niranjan Khadilkar, Keshava Pai K., Thripthi Rai, Vijendra Shenoy, Deviprasad Dosemane

**Affiliations:** 1 Department of Otorhinolaryngology & Head and Neck Surgery, Kasturba Medical College, Mangalore Manipal Academy of Higher Education, Manipal, 576104 Karnataka India; 2 Department of Psychiatry, Kasturba Medical College, Mangalore Manipal Academy of Higher Education, Manipal, 576104 Karnataka India

**Keywords:** Otorhinolaryngology, Depression, Health, Infections, PHQ-9

## Abstract

**Background:**

Depression is a common comorbidity among individuals with otolaryngologic disorders, particularly those with longstanding conditions. This study aims at analysing the sociodemographic profile of depressive disorders in patients with chronic otolaryngology symptoms or conditions, and the correlation with PHQ-9 score.

**Methods:**

A cross-sectional study was conducted on a hundred patients presenting to the outpatient department with chronic otolaryngology symptoms or conditions. They were requested to fill in the PHQ-9 questionnaire, containing questions based on the fourth edition of the *Diagnostic and Statistical Manual of Mental Disorders* (*DSM-IV*) for major depressive disorder (MDD).

**Results:**

Median age was 39, male: female ratio was 1.17. Nasal obstruction (29%), ear discharge (25%), and headache (17%) were the common presenting complaints. Mean and median PHQ-9 scores were 5.03 and 4 respectively. Seven patients (7%) had MDD, while eleven (11%) had other depressive disorder; 9% of cases were found to have no significant otolaryngologic problem despite presenting with symptoms, two of which were found to have depressive disorder. Thirty-five (35%) and thirty-six (36%) patients had minimal and mild depressive symptoms respectively, while one (1%) had severe depressive symptoms. Statistical significance was noted for the duration of symptoms (p-value 0.005); high statistical significance was found for occupation and otolaryngology diagnosis (p-value < 0.001 each). PHQ-9 score showed statistical significance in comparison with gender and duration of symptoms (p-value 0.046 and 0.005 respectively). Correlation of severity of depressive disorder revealed statistical significance with gender (p-value 0.049) and high statistical significance with duration of symptoms (p-value < 0.001).

**Conclusion:**

Chronic otolaryngology conditions are associated with significant morbidity, attributable to longstanding disturbing symptoms and prolonged treatment protocols, leading to depression. Nevertheless, depression in chronic otolaryngology disorders may aggravate or overlap the clinical symptoms or may go undetected. Hence it may be worthwhile to evaluate for depressive disorders in chronic patients presenting to otolaryngology.

## Background

Major depressive disorder represents a significant health concern for the 15–45 age demographic, frequently resulting in considerable disability and reduced productivity. The occurrence of depressive disorders is approximately 5.4–8.9%; it is more frequently seen in patients with prolonged illness (14–75%), thus increasing morbidity as well as mortality and adversely affecting treatment outcomes [1, 2]. Unfortunately, depressive disorders often go undiagnosed by the primary care physician, resulting in greater use of health care funds and facilities, multiple hospital visits, and loss of workdays [3].

Depression is one of the multiple co-morbidities known to be associated with diseases of the ear, nose, and throat (ENT), especially longstanding ones like chronic rhinosinusitis (CRS) and chronic otitis media (COM) [4, 5]. There also exists a solid psychological link between otolaryngologic complaints or conditions such as headache [6], dizziness [7], hearing loss [8], tinnitus [9], allergic rhinitis [10], voice disorders [11], reflux disease [12] and depression (25–36%) [9, 13]. Longstanding symptoms associated with otolaryngologic conditions may precipitate frustration, anxiety, and depression, thus compromising overall mental health and well-being.

Interestingly, nearly 80% of healthy individuals may present with vague symptoms that cannot be medically unexplained, such as tinnitus, giddiness, and headache. The occurrence of such symptoms is 20–27% and 25–40% in primary and secondary care patients respectively, the cause being ambiguous. Although data proves the association between depression and somatisation, the uncertainty about which one happens first remains, as both have similar aetiology, progression, and treatment response [14]. These patients often consult otolaryngologists and the manifestation of depressive disorders in such cases remains unclear.

Given the complexity of symptom presentation, early recognition is essential for effective management. The Patient Health Questionnaire (PHQ-9) is a consistent, authenticated self-assessment used as an instrument for diagnosis, as well as to determine the severity of depressive symptoms [15]. The objectives of this study are to analyse the sociodemographic profile of depressive disorders in patients with chronic otolaryngology symptoms or conditions, and to correlate the same with the PHQ-9 score. A thorough understanding of the relationship between depression and otolaryngology disorders is vital for reducing symptom severity, improving treatment outcomes, and enhancing quality of life.

## Methods

A cross-sectional study was conducted on a hundred patients presenting to the outpatient department with chronic otolaryngology symptoms or conditions. Informed written consent was obtained from the participants. Patients older than 18 were included; patients with symptoms for less than a month, acute infection, and malignancy were excluded. A detailed history was taken, and sociodemographic variables were noted. They were then invited to fill in the PHQ-9 questionnaire, containing questions based on the criteria of the *Diagnostic and Statistical Manual of Mental Disorders* fourth edition (*DSM-IV*) for major depressive disorder. Every question is valued from 0 being *not at all* up to 3 being *nearly every day* and is an indicator of the severity of the disease. A score greater than 10 qualifies for a positive test for depressive disorder. Major depressive disorder (MDD) is diagnosed when five symptoms or more were present for at least more than half of the days in the preceding fortnight, 1 of them being low mood or anhedonia, while other depressive disorder is detected when 2–4 symptoms of depression were present for at least more than half the days in the preceding fortnight, 1 of them being anhedonia or low mood [15]. If found to have a depressive disorder, patients were referred to the Department of Psychiatry for further management. Statistics were analysed with Jamovi software version 2.2.2. P-value below 0.05 was assumed as statistically significant. Clearance was obtained from the Institutional Ethical Committee.

## Results

### Demographics

The study comprised 100 patients, with an age range of 18–75 years, a calculated mean age of 40.56 years, and a median age of 39 years. The gender distribution showed a slight male preponderance, with 54% of patients being men and 46% women, resulting in a male-to-female ratio of 1.17.

### Clinical presentation

Nasal obstruction was the most common symptom at presentation in 29%, followed by ear discharge and headache in 25% and 17% of the cases respectively (Table 1). Duration of symptoms varied from 3 months to 40 years, with a mean duration of 60.07 months.

**Table 1:** Patient characteristics

Patient characteristics	Number (%)
**Age group**	
< 20 years	9 (9%)
21–30 years	25 (25%)
31–40 years	19 (19%)
41–50 years	15 (15%)
51–60 years	20 (20%)
> 60 years	12 (12%)
**Gender**	
Male	54 (54%)
Female	46 (46%)
**Occupation**	
Housewife	31 (31%)
Student	13 (13%)
Labourer	13 (13%)
Self-employed	12 (12%)
Below clerk level	12 (12%)
Clerk level and above	11 (11%)
Skilled professional	7 (7%)
Unemployed	1 (1%)
**Duration of symptoms**	
≤ 6 months	38 (38%)
7–12 months	13 (13%)
13–60 months	27 (27%)
> 60 months	22 (22%)
**Presenting complaint**	
Nasal obstruction	29 (29%)
Ear discharge	25 (25%)
Headache	17 (17%)
Sore throat	14 (14%)
Excessive sneezing	13 (13%)
Hearing loss	7 (7%)
Blocked ear	7 (7%)
Swelling in neck	7 (7%)
Change in voice	5 (5%)
Tinnitus	5 (5%)
Giddiness	4 (4%)
Otalgia	4 (4%)
Oral ulcer	2 (2%)
Epiphora	2 (2%)
Dysphagia	1 (1%)
**Comorbidities**	
Hypertension	7 (7%)
Migraine	7 (7%)
Diabetes mellitus	6 (6%)
Bronchial asthma	3 (3%)
Hypothyroidism	3 (3%)
Human immunodeficiency virus infection	1 (1%)
Chronic liver disease	1 (1%)
Systemic lupus erythematosus	1 (1%)
**Habits**	
Alcohol consumption	16 (16%)
Smoking	7 (7%)
Tobacco chewing	2 (2%)
**Otolaryngology diagnosis**	
Chronic otitis media	26 (26%)
Allergic rhinitis	15 (15%)
Chronic rhinosinusitis	12 (12%)
Deviated nasal septum	7 (7%)
Chronic tonsillitis	6 (6%)
Chronic laryngitis/ laryngopharyngeal reflux	4 (4%)
Multinodular goitre	4 (4%)
Sensorineural hearing loss	4 (4%)
Chronic lymphadenitis	3 (3%)
Vocal cord pathology	3 (3%)
Oral aphthous ulcer	2 (2%)
Chronic dacryocystitis	1 (1%)
Chronic sialadenitis	1 (1%)
Chronic otitis externa	1 (1%)
Temporomandibular joint arthralgia	1 (1%)
Globus pharyngeus	1 (1%)
Nil otolaryngology	9 (9%)
**Diagnosis based on PHQ-9**	
Control	82
Major Depressive Disorder	7
Other Depressive Disorder	11
**Severity of depressive symptoms**	
Nil	14
Minimal	35
Mild	36
Moderate	11
Moderately Severe	3
Severe	1

### Otolaryngologic diagnosis

Chronic otitis media (COM) was the most frequent otolaryngologic diagnosis (26%), followed by allergic rhinitis (15%).

### Screening for depression

**Fig. 1:**
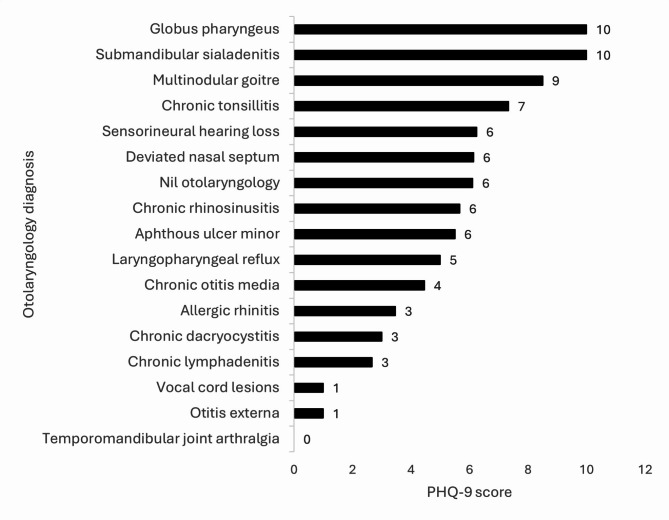
Representation of PHQ-9 score vs. otolaryngology diagnosis

Total PHQ-9 score varied from 0 to 21, with mean and median scores of 5.03 and 4 respectively (Fig. 1). Seven patients (7%) had MDD, while eleven (11%) had other depressive disorder. Nine of the hundred patients (9%) were found to have no significant otolaryngologic problem despite presenting with symptoms. Two of these nine patients were found to have a depressive disorder. Thirty-five (35%) and thirty-six (36%) patients had minimal and mild depressive symptoms respectively, while one (1%) had severe depressive symptoms.

### Statistical analysis

Statistical significance was noted for the duration of symptoms (p-value 0.005); high statistical significance was found for occupation and otolaryngology diagnosis (p-value < 0.001 each) (Table 2**)**. PHQ-9 score was compared with other variables and showed statistical significance with gender and duration of symptoms (p-value 0.046 and 0.005 respectively). Correlation of severity of depressive disorder revealed statistical significance with gender (p-value 0.049) and high statistical significance with duration of symptoms (p-value < 0.001).

**Table 2:** Statistical correlation

**Binomial test**	
Gender	0.484
**χ² Goodness of fit test**	
Age	0.071
Occupation	< 0.001
Duration of symptoms	0.005
Otolaryngology diagnosis	< 0.001
**Mann-Whitney U test**	
PHQ-9 score vs. gender	0.046
**Kruskal Wallis test**	
PHQ-9 score vs. age	0.955
PHQ-9 score vs. occupation	0.6
PHQ-9 score vs. duration of symptoms	0.005
PHQ-9 score vs. otolaryngology diagnosis	0.22
**Chi-square test**	
Depressive disorder vs. age	0.1
Depressive disorder vs. gender	0.664
Depressive disorder vs. occupation	0.58
Depressive disorder vs. duration of symptoms	0.219
Depressive disorder vs. otolaryngology diagnosis	0.205

## Discussion

Depression occurs due to abnormal regulation of the brain, increasing physical as well as the psychological burden of symptoms [2]. Clinicians must not inevitably assume that depressive disorders are an independent entity in patients with chronic disease, as they may be interlinked, thus requiring a more comprehensive management strategy. Psychiatric or psychological comorbidities can modify symptom perception in chronic disease, due to the negative impact on the central nervous system through the hypothalamic-pituitary-adrenal axis releasing cytokines and leading to chronic inflammation [16]. Depression is considerably underdiagnosed in primary healthcare and general hospital setups; 73.5% of patients with major depressive disorders present with somatic, not psychiatric, symptoms, this is referred to as *masked depression* [3]. Even in non-surgical conditions, depressive disorders predict poor outcomes [2]. Higher levels of depression are linked to exaggerated pain perception and mood changes, which is the result of abnormal activity of the anterior cingulate cortex and insula, as identified by functional magnetic resonance imaging scanning [17].

In the present study, patients were equally distributed across all age groups. The younger population had lower scores (mean PHQ-9 score of 4.68 and 5.89 in 21–30 years and 31–40 years age groups respectively), whereas the highest severity of depressive symptoms (mean PHQ-9 score of 6.07) was noted in patients with the age range of 41–50 years. This finding suggests that middle-aged individuals may be more vulnerable to depression due to health concerns, increasing professional responsibilities, and family problems. The pressures of establishing a career, raising a family, and managing financial obligations may contribute to enhanced stress levels and ensuing depressive symptoms. Litvack and co-authors found 25% of the patients to have depression at an age averaging 46.7 years [1]. A study by Cheng and co-authors to correlate depression and dizziness revealed slightly higher scores in the younger population compared to the elderly (6.74 ± 5.31 vs. 6.19 ± 4.28 respectively); however, it was not statistically significant [18].

A slight male preponderance (54%) was noted in the current study. However, the severity of depressive disorder was detected to be higher in women than in men (mean PHQ-9 score of 5.76 vs. 4.41 respectively). There exists a social stigma toward seeking mental healthcare; lack of family support and hormonal alterations add to the increased disease burden in women. Three cases of MDD were seen in men, two cases were noted in women, while other depressive disorder was seen in three men and eight women. This is comparable to the results of Mace et al.; subjects with depression were more likely to be females than patients not having depression (p-value 0.002). Occurrence of depression is almost twice as common in women, and in people with comorbidities (14–75%) than in the general population [2].

The majority of the patients in the present study were housewives (31%), followed by students and labourers (13% each). The severity of depressive symptoms was noted to be the highest in the unemployed, followed by skilled professionals and housewives (mean PHQ-9 scores of 7, 6.86, and 5.65 respectively). Two cases each of MDD were noted in the skilled professionals, housewives, and below-clerk categories. Four of the seven cases of other depressive disorder were seen in housewives. Monetary disconcertment, lifestyle factors, stress, lack of socialisation, and caregiving responsibilities contribute to the manifestation of depressive disorders in these patients. Nevertheless, statistical significance was not noted between occupation and depressive disorder or severity of depressive symptoms (p-value 0.58 and 0.6 respectively).

Most of the patients in our study (38%) presented within six months of the onset of otolaryngology symptoms; four patients with other depressive disorder belong to this category. Two cases of MDD were noted in patients with symptoms between 7 and 12 months and 13–60 months each. Nonetheless, the severity of symptoms was highest in patients who presented between 7 and 12 months of onset of otolaryngology symptoms (mean PHQ-9 score of 6.62), followed by patients who presented beyond 5 years of onset of symptoms (mean PHQ-9 score of 6.33). Statistical significance was found in correlating the duration of symptoms with the severity of depressive symptoms (p-value 0.005). Depressive disorders in longstanding illnesses may be due to increased consumption of healthcare resources, increased physician visits, missed days of work, and greater use of antibiotics [1].

In our study, minimal to mild depression was seen in 80% of the subjects, which compared well with Litvack et al., who found only 25% of patients with moderate or severe depression as per the PHQ-9 survey [1]. Nanayakkara concluded that mental health is associated with a patient’s subjective symptom scores [19]. Independent t-test was used to correlate PHQ-9 score in patients with mild depression, MDD, and other depressive disorder, and was of statistical significance (*p* = 0.022).

Patients with hidden depressive disorder and somatic symptoms often visit physicians instead of a psychiatrist, thus utilizing greater healthcare resources [3]. Somatic symptoms unelucidated by physical examination are described as medically unexplained symptoms (MUS), which comprise giddiness, and tinnitus. MUS are frequent, with varied presenting features, accounting for up to 45% of all general practice consultations, and without a clear diagnosis at 3 months in as high as 50% of patients [20]. Goto found MUS in 9.2% (90/983) of the patients visiting the otolaryngology department; 5.0% (49/983) had MUS with depression, while 4.2% (41/983) had MUS without depression [3]. MUS may be classified (a) based on DSM-IV criteria, (b) those without a DSM-IV diagnosis, or (c) medical MUS syndromes [21]. In this study, nine patients (18%) had no significant otolaryngology disease, indicating that their symptoms were merely a result of underlying mood disorders. Tinnitus, giddiness, and throat irritation were the commonest MUS in these patients (3 cases each). Three of the nine patients (6% of all cases included in the study) were found to have primary depression; secondary depression was noted in fifteen (30%) patients. Although comorbid depressive disorders are probably bidirectional and have a worse prognosis, it is challenging to determine the causality [22]. It is unclear whether presenting symptoms contribute to depression or vice versa [17]. Identifying underlying depression in such patients impacts patient management; emphasis on symptom management, mood stabilisation, behavioural interventions, and lifestyle modifications may be required in addition to integrated team approach, pharmacotherapy, and psychotherapy.

Wang and co-authors performed a systematic review and meta-analysis to estimate the prevalence of depression and depressive symptoms in outpatients; they found the highest prevalence of depressive disorders and symptoms in otolaryngology patients (53%), followed by dermatology and neurology (39% and 35%) respectively [23]. The occurrence of depressive disorder was noted to be 36% in the present study. Chronic otitis media was the most frequent otolaryngology diagnosis in the present study (26%). However, only 1 of the 26 patients (0.04%) had major depressive disorder. Allergic rhinitis was the next most common otolaryngology disorder (15%); none of these patients were found to have depression. A possible cause of disparity between the sinonasal symptoms and the actual infection is the existence of psychiatric comorbidities, which may alter clinical presentation [16]. Neuroticism (emotional instability) can stimulate somatic sensations like nasal obstruction and correlates well with tiredness, depression, and autonomic pupillary disturbances [24]. Nanayakkara found a significant association between sinonasal symptoms and depression score (*p* = 0.02) [19]. The severity of depression symptoms was the greatest in patients with chronic tonsillitis, followed by sensorineural hearing loss and deviated nasal septum (PHQ-9 scores of 7.33, 6.25, and 6.14 respectively). Brewster found that age-related hearing loss was associated with a greater likelihood (1.63 times) of having depressive symptoms compared to a healthy population, and it was statistically significant [25]. Litvack and co-authors concluded that patients with depressive disorders have a worse quality of life, even while other indicators of the severity of the disease may be similar; an increase in pain perception, oropharyngeal and CRS symptoms, lethargy, difficulty in daily chores and occupation has been reported [1]. Interestingly, three of the four patients with colloid goitre were found to have depression (one case of MDD and two cases of other depressive disorder). Mood disorders are known to have a greater prevalence in thyroid dysfunction [26]; nevertheless, all four patients were clinically euthyroid. Chandra in his study concluded that a greater rate of depression is seen in chronic otolaryngology disorders (10-14%) compared to that in a similar age group among the general population (7.3%). This was the maximum in patients with inner ear pathology, and sleep apnoea [27]. Our study encompasses a variety of chronic otolaryngology conditions, while a lot of previous studies have focused exclusively on individual otolaryngology diseases. However, the small number of subjects included in the study is a limitation. Further research is required to assess the complex association of depression and otolaryngologic conditions and symptoms in larger populations, to evaluate the temporal and causal relationship and the efficacy of integrated mental health interventions in otolaryngology practice.

Patients with inexplicable complaints are habitually subjected to multiple diagnostic tests and interventions, which may not reveal organic disease, and might be considered pointless in retrospect, except that they eliminate known medical conditions. The price of somatisation to healthcare services is high. Additionally, the impact on a patient’s quality of life can be grave. Socioeconomic status, literacy, culture, and childhood experiences, all affect the degree to which emotional suffering is expressed as physical symptoms [14]. The association between otolaryngology conditions and psychology is snowballing swiftly due to greater awareness among populations. Patients browse for disease-related information and become uneasy and distressed; some patients become over-anxious about mild symptoms such as nasal stuffiness or discharge, a lump in the throat perceived as cancer. Understanding the patient psyche and reassurance goes a long way in allaying suffering, along with medical or surgical treatment [28]. Chronic otolaryngology patients should be screened for depressive disorder, and offered education, counselling and integrated treatment approaches, including cognitive behavioural therapy, stress reduction techniques, to improve quality of life and overall wellbeing [14].

## Conclusion

This study highlights the critical link between chronic otolaryngology disorders and depressive disorders, emphasizing the necessity of integrating mental health evaluation into otolaryngology practice. Chronic conditions of ear, nose, and throat are associated with significant morbidity, attributable to longstanding disturbing symptoms, and prolonged treatment protocols. This may result in the manifestation of depressive disorders. Nevertheless, the presence of depressive disorders in chronic otolaryngology disorders may aggravate or overlap the clinical symptoms, and depression may go undetected. Hence it is worth evaluating for depression in chronic patients presenting to otolaryngology. Timely intervention improves the presenting condition and, even so, uplifts patients from their psychological suffering, thus assuring a better quality of life. Furthermore, the presence of this comorbidity has significant implications on healthcare policymaking, resource allocation, and research, leading to better patient outcomes.

## Data Availability

No datasets were generated or analysed during the current study.
